# Efficacy and Safety of Intravitreal Fluocinolone Acetonide Implant for Chronic Diabetic Macular Edema Previously Treated in Real-Life Practice: The REALFAc Study

**DOI:** 10.3390/pharmaceutics14040723

**Published:** 2022-03-28

**Authors:** Thibaud Mathis, Maxence Papegaey, Cécile Ricard, Amina Rezkallah, Frédéric Matonti, Aditya Sudhalkar, Cristina Vartin, Corinne Dot, Laurent Kodjikian

**Affiliations:** 1 Service d’Ophtalmologie, Hôpital de la Croix-Rousse, Hospices Civils de Lyon, 69004 Lyon, France; m.papegaey@gmail.com (M.P.); amina.rezkallah@chu-lyon.fr (A.R.); christine.vartin@chu-lyon.fr (C.V.); 2 UMR-CNRS 5510 Matéis, Université Lyon 1, 69100 Villeurbanne, France; 3 StatMed74, 74000 Annecy, France; stat.med74@gmail.com; 4 Centre Monticelli Paradis, 13008 Marseille, France; frederic.matonti@free.fr; 5 Institut Neuroscience Timone, Univeristé Aix Marseille, CNRS, INT, 13005 Marseille, France; 6 Groupe Almaviva Santé, Clinique Juge, 13008 Marseille, France; 7 Alphavision Augenzentrum, 27568 Bremerhaven, Germany; adityasudhalkar@icloud.com; 8 Service d’Ophtalmologie, Hôpital Militaire Desgenettes, 69003 Lyon, France; corinnedot.pro@hotmail.fr; 9 Hôpital D’instruction des Armées, Val-de-Grâce, 75005 Paris, France

**Keywords:** corticosteroids intravitreal injection, diabetic macular edema, diabetic retinopathy, fluocinolone acetonide implant

## Abstract

Diabetic macular edema (DME) is the main cause of visual impairment in diabetic patients and a chronic disease requiring long-term treatments. The fluocinolone acetonide (FAc) implant has recently been approved to treat DME in patients considered insufficiently responsive to available therapies. This study evaluates the functional and anatomical efficacy of the FAc implant in real-life practice. A total of 62 eyes with chronic DME were included and followed for a mean of 13.9 (+7.5) months. Previous treatment included at least anti-vascular endothelial growth factor (VEGF) in 83.9% of eyes, dexamethasone implant (DEX-I) in 100% of eyes, vitrectomy in 29.0% of eyes, and laser photocoagulation (either panretinal or focal photocoagulation) in 75.8% of eyes. The mean baseline best corrected visual acuity (BCVA) was 64.0 (+/−17.2) letters (median: 67.5 letters) with a mean DME duration of 60.3 (+/−30.6) months. The maximum BCVA gain occurred at 21 months with a mean gain of 5.0 (+/−12.7) letters. A total of 50.0% of eyes gained ≥5 letters during follow-up. Patients with lower BCVA at baseline had the lowest final BCVA (*p* < 0.001) but the highest BCVA gain (*p* = 0.02). The best overall improvement in mean central macular thickness (CMT) occurred at 18 months (*p* < 0.0001). The improvement in BCVA was inversely associated with the decrease in CMT and showed a decrease when CMT increased (DME recurrence). According to the history of vitrectomy, we did not find any significant difference in mean final BCVA (*p* = 0.1) and mean BCVA gain (*p* = 0.2) between eyes previously vitrectomized or not. A total of 23 eyes (37.1%) required additional treatment for DME, and 17.7% required an IOP-lowering procedure during follow-up. In conclusion, this real-life observational study demonstrated the efficacy and safety of the FAc implant in patients with chronic DME already treated with other available therapies.

## 1. Introduction

Diabetes Mellitus (DM) was estimated to affect a total of 463 million people worldwide in 2019 and prevalence is set to rise to around 700 million by 2045 [1]. Diabetic macular edema (DME) is the main cause of visual impairment in diabetic patients, with a prevalence of 7.6% in the Diabetic Retinopathy Barometer study [2]. Without treatment, nearly half of patients with DME will lose two or more lines of visual acuity within 2 years [3]. Current guidelines for the first-line treatment of DME consist of between three and five monthly intravitreal injections of anti-vascular endothelial growth factor (VEGF) [4]. However, despite promising results in randomized controlled studies, poorer outcomes in terms of visual improvement have been reported in observational studies. This can be explained by the lower number of injections received in real-life practice in comparison to clinical trials [5,6]. Moreover, it is well-known that up to 30–40% of patients have an inadequate response to anti-VEGF treatment [7], probably due to a lower concentration of VEGF in certain patients’ eyes [8]. Steroids are another therapeutic option for treating DME by decreasing the pro-inflammatory cascade that leads to DME [9]. Dexamethasone-implant (DEX-I) was the first steroid approved by FDA for DME and can be used as first- or second-line therapy [10]. Its efficacy in terms of visual acuity in DME patients has been demonstrated in numerous studies and it also dramatically reduces the treatment burden in routine practice, with injections required every 4–6 months [6]. However, as a chronic disease, DME requires long-term treatment and even patients under DEX-I need to be regularly reinjected [11,12]. The fluocinolone acetonide (FAc) implant, delivering 0.2 µg/day in the vitreous, is another steroid recently approved for the treatment of visual impairment in chronic DME patients considered insufficiently responsive to available therapies such as anti-VEGF or DEX-I. It has shown favorable outcomes in randomized controlled trials with visual improvement at 3 years for patients already treated with other therapies [13]. However, as it is a recent molecule, little is known about the efficacy and safety of this treatment in routine practice.

The primary objective of our study is to evaluate the functional and anatomical efficacy of the FAc implant in real-life practice. The secondary objectives are to determine prognostic anatomical biomarkers of treatment response, the need for additional treatment, and the safety profile.

## 2. Materials and Methods

### 2.1. Study Design and Population

We conducted a retrospective bicentric study at the Croix-Rousse University Hospital and Desgenettes Military Hospital in Lyon, France. We included consecutive patients who received an FAc implant for DME between December 2018 and July 2021. Inclusion criteria were patients with type 1 or type 2 DM, aged ≥ 18 years, with chronic DME previously treated with DEX-I or anti-VEGF injections, who were then injected with an FAc implant. Patients receiving FAc implants bilaterally were also eligible for inclusion. Patients with another associated cause of cystoid macular edema were excluded, including uveitis, retinal vein occlusion, or postoperative edema. The study was conducted in accordance with the Declaration of Helsinki. An international review board approved the study (Ethics Committee of the French Society of Ophthalmology, IRB 00008855 Société Française d’Ophtalmologie IRB#1). All included patients received written information about the study and gave their consent for the study.

### 2.2. Data Collection

Patient demographic, medical, and ophthalmological data were collected retrospectively from patient records, and included age, gender, duration of DME, ophthalmological history, lens status, previous DME treatments, and presence of panretinal photocoagulation (PRP). A complete ophthalmological examination including measurement of best corrected visual acuity (BCVA) on the early treatment diabetic retinopathy study (ETDRS) scale, intraocular pressure (IOP), slit lamp examination, fundus examination, and spectral domain optical coherence tomography (SD-OCT, Cirrus HD-OCT; Carl Zeiss Meditec, Dublin, CA; Spectralis OCT; Heidelberg Engineering, Heidelberg, Germany) was performed at the initial visit and repeated at each follow-up visit for all patients, using the same SD-OCT device. The central macular thickness (CMT) was automatically calculated by the SD-OCT software and manually adjusted when necessary. The following SD-OCT biomarkers were collected at baseline, within the central 1 mm area: (i) presence of subretinal fluid (SRF); (ii) presence of intraretinal fluid (IRF); (iii) presence of hyperreflective foci (HRF), defined as hyperreflective dots <30 µm, punctuated with a reflectivity similar to that of the nerve fiber layer; (iv) disorganization of the retinal inner layers (DRIL); (v) alteration of the ellipsoid/interdigitation zone (EZ/IZ), defined by a clear discontinuity in these layers; (vi) presence of hard exudates.

### 2.3. Treatment Initiation and Follow-Up

According to the most recent international guidelines, treatment with FAc can be introduced shortly after anti-VEGF or DEX-I injections [14]. Patients were then monitored every 3 months in the absence of any additional treatment. If additional anti-VEGF treatment was started, patients were generally monitored monthly. If additional DEX-I treatment was started, follow-up was generally shortened to every two months according to national guidelines [15].

The FAc implant was injected through the pars plana using a 25-gauge injector. Each injection was performed after the patient was appropriately informed. Additional treatment with DEX-I or anti-VEGF (ranibizumab or aflibercept), or focal laser was decided on at the ophthalmologist’s discretion, and generally administered in the event of anatomical DME recurrence and/or a significant drop in VA.

### 2.4. Outcome Measures

The primary outcome measure was the mean change in BCVA using the ETDRS scale between the last visit before FAc injection (defined here as baseline) and the last follow-up visit. Secondary outcome measures included the mean change in BCVA according to the baseline BCVA subgroup (<50 L, 50–60 L, >60 L), according DME duration subgroup (<24 months, 24–48 months, >48 months), according to baseline CMT subgroup (<300 µm, 300–400, ≥400 µm), the proportion of eyes with significant functional response (i.e., defined as a BCVA gain ≥ 5 letters), the mean CMT change between baseline and the last follow-up visit, the proportion of eyes with significant anatomical response (i.e., defined as a decrease in CMT ≥ 20%), the need for additional treatment, and the occurrence of any adverse events. For IOP outcomes, we defined ocular hypertension (OHT) as IOP > 25 mmHg or an increase in IOP ≥ 10 mmHg after FAc treatment.

### 2.5. Statistics

Qualitative variables were recorded as numbers and percentages, quantitative variables as the mean, standard deviation (SD), median, and range. BCVA, CMT, and IOP are expressed as values at each follow-up visit or as a variation from baseline. The clinical data at 1 month correspond to the last values recorded at 1 month ± 15 days. For the 3-month follow-up, the BCVA, CMT, and IOP values retained are the last values recorded between 1.5 months and 4.5 months. The same rule (±1.5 months) was applied for the subsequent follow-up steps (every 3 months). Comparisons between the clinical data at different follow-up stages and the baseline values were made using Student’s *t*-tests on matched data when the number of patients allowed it and using Wilcoxon tests on linked samples if this was not the case. For the quantitative variables, the comparisons between different groups were made using Student’s *t*-test or Mann–Whitney (or Kruskal–Wallis) test if required. For the qualitative variables, the comparisons between groups were made using Chi2 or Fisher tests where the numbers allowed. Kolmogorov–Smirnov tests were used to compare the VA distributions according to different groups. All analyses were performed with SPSS version 27 software. The significance level was set at 0.05.

## 3. Results

### 3.1. Patient Population

A total of 62 eyes in 46 patients with chronic DME treated with FAc implants were included in the study. The mean (SD) age of the patients was 71.6 (8.9) years, and 84.8% had type 2 diabetes, with a mean (SD) disease duration of 25.0 (12.1) years and a mean (SD) DME duration of 60.3 (30.6) months. Only two eyes (3.2%) were phakic at baseline, and 18 (29.0%) had a previous history of vitrectomy (all without silicone oil). Twenty eyes (32.3%) had a history of IOP-lowering medication or previous IOP-lowering surgery at baseline. Fifty-two eyes (83.9%) had received previous anti-VEGF and 100% had received previous DEX-I injections for DME. The last treatment before FAc was anti-VEGF in 8 eyes (12.9%) and DEX-I in 54 eyes (87.1%), with a mean (SD) delay between last treatment and FAc of 1.7 (1.6) months and 3.8 (2.6) months, respectively. The mean (SD) duration of follow-up after FAc was 13.9 (7.5) months, and 38 (61.3%) eyes had a follow-up ≥12 months (Table 1). Ten eyes (16.1%) were lost to follow-up: three were lost to follow-up after their first visit after FAc, five were lost to follow-up after the second, and two were lost to follow-up after the third.

### 3.2. Functional Outcomes

**Figure 1:**
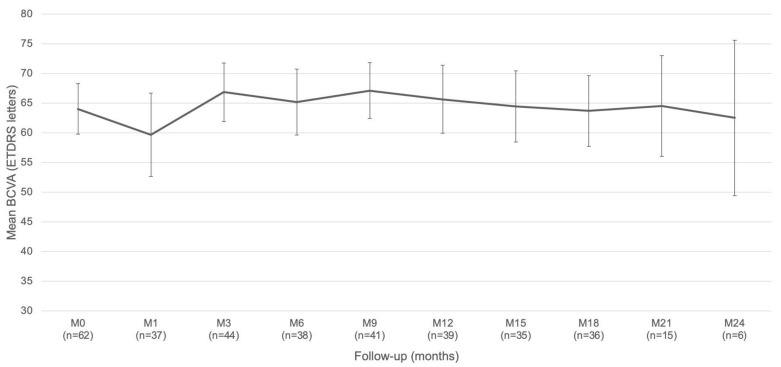
Mean change in best corrected visual acuity (BCVA).

The mean (SD) baseline BCVA was 64.0 (17.2) letters (median: 67.5 letters). The maximum mean (SD) BCVA was 66.8 (16.7) letters, observed at 3 months, and was significantly different from baseline (*p* = 0.03, Figure 1).

**Figure 2:**
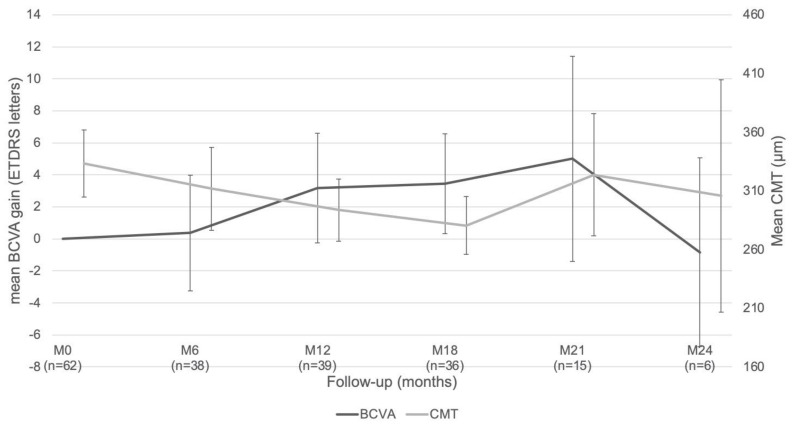
Mean best corrected visual acuity (BCVA) improvement and mean change in central macular thickness (CMT).

The maximum BCVA gain occurred at 21 months, with a mean (SD) gain of 5.0 (12.7) letters (Figure 2). Overall, 31 eyes (50.0%) were functional responders, 18 eyes (29.0%) had a ≥ 10-letter improvement, and 12 eyes (19.4%) had a ≥ 15-letter improvement during the follow-up period.

According to baseline BCVA, patients with BCVA < 50 letters had the lowest BCVA at the end of the follow-up (*p* < 0.001) but the highest BCVA gain (*p* = 0.02). According to DME duration, there was a significant difference in BCVA at the end of the follow-up period (*p* = 0.004), but not in BCVA gain (*p* = 0.5). In terms of DME thickness, there was no significant difference in BCVA at the end of the follow-up period (*p* = 0.1), however, there was a significant difference in BCVA gain (*p* = 0.01). Regarding the history of vitrectomy, there was no significant difference in BCVA at the end of the follow-up period nor in BCVA gain (*p* = 0.1 and *p* = 0.2, respectively). When considering the presence of SD-OCT biomarkers, there was a significant difference in final BCVA in the presence of HRF (*p* = 0.01) and EZ/IZ alterations (*p* < 0.001), and there was a trend for DRIL (*p* = 0.09). However, none of these SD-OCT biomarkers were significantly associated with BCVA gain, excepted for the presence of SRF (*p* = 0.048, Table 2).

### 3.3. Anatomical Outcomes

The mean (SD) baseline CMT was 333.3 (112.6) µm, and the mean (SD) CMT at 24 months was 305.7 (121.4) µm. The best overall mean (SD) CMT improvement occurred at 18 months and was significantly different from baseline at 280.5 µm (74.4) (*p* < 0.0001, Figure 2). A total of 23 eyes (37.1%) were considered as anatomical responders. For patients with an initial CMT < 300 µm (mean (SD) CMT: 246.6 (35.3) µm at baseline) the mean CMT consistently remained below the 300 µm threshold throughout follow-up, with a significant minimum mean (SD) CMT of 239.0 (59.3) µm at 18 months in comparison to the baseline value (*p* < 0.001). Eyes with an initial CMT ≥ 300 µm (mean (SD) CMT: 400.1 (105.8) µm at baseline) showed a significant minimum mean (SD) CMT of 310.1 (70.8) µm at 18 months in comparison to the baseline value (*p* = 0.04).

### 3.4. Need for Additional Treatment for DME

A total of 23 eyes (37.1%) required additional treatment for DME. Of these eyes, 11 (47.8%) received intravitreal aflibercept, 15 (65.2%) received DEX-I, 12 (52.2%) received focal macular laser, and 2 (8.7%) received a second FAc injection (13.1 and 19.8 months after the first FAc injection). The mean (SD) time to the introduction of an additional therapy was 7.2 (4.7) months. The mean (SD) number of additional treatments was 1.6 (3.0) for the whole population and 4.4 (4.2) considering only the retreated population. Four eyes had eight or more additional treatments (range: 8–18). The 19 remaining eyes had a mean (SD) of 2.9 (2.0) additional treatments per eye during the follow-up period. The mean (SD) frequency of post-FAc treatment was one treatment every 10.3 (5.8) months in comparison to 3.7 (9.9) months before FAc injection in the present cohort.

**Figure 3:**
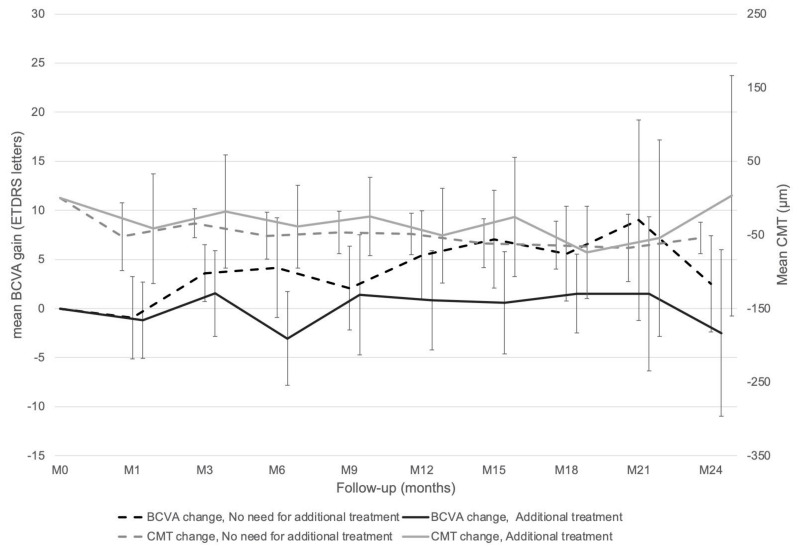
Best corrected visual acuity (BCVA) and central macular thickness (CMT) evolutions depending on need for additional treatment or not.

The comparison between eyes which did not need additional treatment and those which did showed a non-significant trend for higher mean (SD) final BCVA (68.4 (16.9) letters versus 62.3 (15.2) letters, respectively, *p* = 0.06) and a significant difference for higher mean (SD) final CMT (270.5 (83.0) µm versus 318.6 (88.3) µm, respectively, *p* = 0.02, Figure 3) in favor of the group without additional treatment.

Amongst the different patient characteristics at baseline, only the presence of PRP was found to be associated with the need for additional treatment (*p* = 0.01, Table 3).

### 3.5. IOP Outcomes

**Figure 4:**
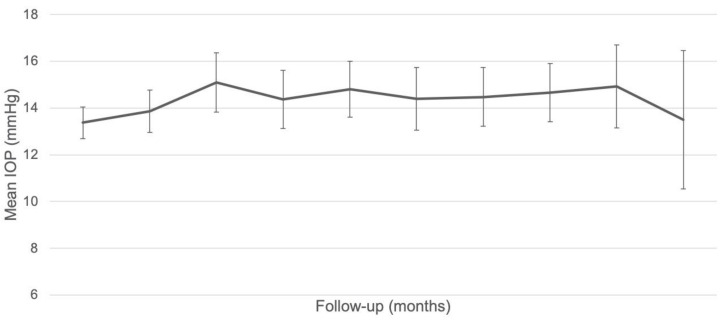
Change in intraocular pressure (IOP) throughout follow-up.

The mean (SD) baseline IOP was 13.4 (2.7) mmHg. The mean (SD) IOP increase during follow-up was +1.8 (4.1) mmHg at 3 months (*p* = 0.005), +1.3 (4.1) mmHg at 6 months (*p* = 0.06), +1.7 (3.8) mmHg at 9 months (*p* = 0.007), +1.5 (4.0) mmHg at 12 months (*p* = 0.03), +1.6 (3.1) mmHg at 15 months (*p* = 0.003), +1.7 (3.4) mmHg at 18 months (*p* = 0.005), +1.7 (2.9) mmHg at 21 months (*p* = 0.04), and +1.7 (2.7) mmHg at 24 months (*p* = 0.2, Figure 4).

There was no difference in final mean (SD) IOP between patients naïve to IOP-lowering therapy and those previously treated with IOP-lowering therapy (12.5 (4.9) mmHg versus 14.0 (3.6) mmHg, respectively *p* = 0.8). At baseline, 11 eyes (17.7%) were on monotherapy, 10 eyes (16.1%) were on dual therapy, and 1 eye had a history of IOP-lowering surgery. A total of six eyes (9.7%), had an increase in IOP > 21 mmHg during follow-up and seven eyes (11.3%), had OHT. Eleven eyes (17.7%) required an IOP-lowering procedure during follow-up, including additional IOP-lowering therapy, selective laser trabeculoplasty (SLT), or surgery. The mean (SD) time to introduction/change of IOP-lowering therapy was 12.9 (8.8) months. For the four eyes that received post-FAc SLT, the mean (SD) time to SLT was 14.6 (10.2) months. Only one eye underwent IOP-lowering surgery during follow-up, 9.2 months after FAc implant and despite treatment with an IOP-lowering therapy (Table 4). The mean total number of injections before FAc was significantly higher in the OHT eyes compared to the 55 remaining eyes (23.4 (9.8) injections versus 14.9 (8.4) injections, respectively, *p* = 0.03): the mean (SD) number of anti-VEGF injections did not differ significantly (11.0 (8.1) injections versus 8.9 (5.7) injections, respectively, *p* = 0.5), but the mean (SD) number of DEX-implant IVT before FAc was significantly higher in the group with OHT (12.4 (4.2) injections versus 7.6 (3.9) injections, respectively, *p* = 0.01).

### 3.6. General Safety

For the two phakic eyes included at baseline, cataract surgery was performed 250 days and 251 days after FAc implantation. For these eyes, an additional injection of DEX-I was administered at the end of the surgery. Concerning the whole cohort, no anterior chamber migration of the FAc implant was observed and no endophthalmitis or vasculitis occurred following FAc implantation.

## 4. Discussion

This real-life observational study demonstrated the efficacy and safety of the FAc implant in the treatment of chronic DME, thus confirming the results of previous randomized controlled studies of this molecule. This study showed an improvement in mean BCVA which started at 3 months and remained stable during the follow-up period. The strongest baseline characteristic associated with final BCVA and BCVA gain was baseline BCVA: poor baseline BCVA was associated with worse final BCVA, but better BCVA gains. This association has already been demonstrated in other studies and reviews of DME and should prompt the early treatment of eyes with macular disease, before they experience severe visual loss [16,17]. This is also underlined by the better final BCVA for eyes with a short duration of DME [17]. These functional results were associated with anatomical improvement, demonstrated as a decrease in CMT from baseline throughout the follow-up period. It should be noted that the mean CMT decreased continuously until 18 months, but with a subsequent increase in thickness at 21 months due to edema recurrence. Regarding BCVA gain, the improvement in BCVA was inversely associated with the decrease in CMT, showing a continuous increase until 21 months followed by a decrease in BCVA gain. This correlation between an initial decrease in CMT, followed by BCVA gain, has already been described, and confirms that DME treatment should be initiated on anatomic recurrence, without waiting for any functional deterioration [18]. Other SD-OCT biomarkers have also been found to be associated with a worse visual prognostic herein, confirming the findings of previous studies in the literature. The presence of HRF, DRIL, and EZ/IZ alterations have already been described as associated with the lowest final BCVA [19,20], and the presence of SRF as associated with the highest BCVA gain [21]. Moreover, it has been shown that some SD-OCT biomarkers, such as the presence of SRF, HRF, and hard exudates, are predictors of a better response to steroids in comparison to anti-VEGF. Therefore, an SD-OCT-based decision has been included in the recent algorithm for DME management [10]. Finally, and similar to what has already been found with DEX-I [22,23], we did not find any significant difference in functional effectiveness according to the history of vitrectomy.

At first glance, our present results could be considered to be disappointing in comparison to other real-life studies showing a mean visual gain of more than eight letters and with more than 85% of eyes showing a functional response to FAc [17]. However, it should be noted that the baseline characteristics of eyes included in these studies showed lower baseline BCVA and higher CMT than our series (mean of 64 letters and median of 67.5 letters herein) [24,25,26,27,28,29,30,31]. This is explained by the inclusion of patients in these other studies who were probably experiencing significant edema recurrence at baseline. Unlike previous reports in the literature, we aimed to use FAc to treat cases of DME which were already well-controlled by a previous intravitreal therapy with the aim of reducing the treatment burden. This is emphasized by the delay between the last injection before FAc and FAc treatment, which is approximately 1.5 months for anti-VEGF and less than 4 months for DEX-I. In this paradigm, the ultimate goal of the treatment is to keep the macula dry, avoiding fluctuations in macular thickness, which are known to be associated with poorer visual outcomes in eyes with DME [32,33]. In comparison to FAc, DEX-I shows higher water solubility and therefore requires the use of a sustained-release implant to increase its durability into the vitreous. DEX-I measures 7 × 0.37 mm and contains 700 µg of dexamethasone. It is composed of poly (lactic acid-co-glycolic acid) polymers that degrade into carbon dioxide and water as dexamethasone is released. Once injected, the implant delivers a “bolus” of dexamethasone into the eye (peak of 1110 ng/g observed at 8 weeks post-injection) and then a decrease over 6 months [34]. In comparison, the FAc implant is a nonbiodegradable polyimide cylinder measuring 3.5 × 0.37mm filled by a polymer matrix loaded with fluocinolone acetonide. It is designed to deliver lesser concentrations of steroids in the first few days following the injection (1.26 ng/g), but with a sustained release of 0.2 µg per day for a 36-month period [35]. Due to its pharmacokinetics, an FAc implant should not be considered as a “bolus” treatment but rather as a “basal” therapy that helps stabilize the disease when injected, preferably in patients with DME which is responsive to steroid therapy. This new treatment paradigm, used in the present cohort and highlighted in the recent consensus guidelines on the use of FAc [14], is the originality and strength of our study.

In our study, despite the inclusion of patients with chronic DME, we showed that almost two-thirds of patients did not need additional therapy over the course of the follow-up period. Most importantly, the frequency of retreatment dramatically decreased from 3.7 months to 10.3 months after FAc injection, similar to the findings of the USER study retrospectively analyzing charts from US centers [36]. Moreover, it should be noted that eyes which did not require additional treatment logically had better functional and anatomical results, although the results for BCVA did not reach the significance threshold. A retreatment decision cannot be defined as a failure of FAc if the delay between FAc injection and retreatment decision is longer than the delay between two treatments before FAc. Unlike DEX-I, FAc continuously delivers a very low dose of corticosteroids into the vitreous and retina, allowing for sustained release over a long period of time, between 18 and 36 months according to the different clinical studies and manufacturers [37]. This allows the molecule to impregnate the retina in the long term, making it particularly suitable for the treatment of chronic macular diseases such as DME. However, it seems that some eyes require a higher dose of corticosteroids to maintain a dry retina, justifying additional treatments, as a “bolus” therapy. Therefore, the effectiveness of FAc injection should in this case be evaluated in a risk/benefit ratio with the patient, taking into account the reduction in the treatment burden, the adverse events of the injection/molecule, and the coast of the treatment.

Regarding safety, our findings show that the FAc implant was generally well tolerated during follow-up. It is well known that steroids can induce OHT and glaucoma. However, despite the inclusion of almost one-third of eyes with a history of IOP-lowering procedures, only 11% of the eyes analyzed experienced OHT during follow-up, and 17.7% required an IOP-lowering procedure, including one eye which required surgery. The results regarding changes in IOP and its management appear to be poorer than those reported in randomized controlled trials, but in the same proportion as those reported in real-life studies [17,38]. Interestingly, we found that the number of DEX-I injections before FAc was significantly higher in the group with OHT, but no conclusions can be drawn based on this secondary analysis due to the small number of patients. These data suggest a cumulative effect of steroids on the incidence of OHT, but it is not clear whether this risk of OHT is associated with the high number of DEX-I injections before FAc or the duration of steroid exposure. However, this cumulative effect was not demonstrated in the SAFODEX-2 study, which reported on the long-term safety of DEX-I in the treatment of macular edema due to diverse causes [12]. As expected, the two phakic eyes treated in the present study underwent cataract surgery during follow-up, and it was decided to inject DEX-I as an additional therapy at the end of surgery to prevent any DME recurrence that might be triggered by the surgery [39].

We acknowledge several limitations to the present study. Firstly, its retrospective nature limits the collection and the exhaustivity of the data analyzed. Moreover, unlike randomized controlled trials, observational studies do not have strict protocol with precise follow-up time and retreatment criteria. The population included is also inhomogeneous and different factors can influence the outcomes. However, this is generally the case for real-life studies, which are essential for adapting a treatment to general routine practice [6,17,40]. Secondly, the relatively low number of patients included limits the significance of the results and means its conclusions cannot be applied to other centers. This is a common bias in reports studying novel therapeutic methods, especially when indicated as second- or third-line therapies, as it was suggested for FAc. Finally, the follow-up period could be considered as short for a chronic macular disease, but more than 60% of patients were followed more than one year, which is longer than most real-life studies.

In conclusion, this study shows that FAc implants are a well-tolerated treatment option to reduce the therapeutic burden in DME, bearing in mind that patients with diabetes also have to attend many consultations with other specialists [41]. This novel therapeutic option can be proposed to patients who responded well to DEX-I or anti-VEGF treatments, with the aim of, firstly, limiting injections and visits, secondly, preventing DME recurrence, and thirdly, reducing anatomical fluctuations.

## Figures and Tables

**Table 1:** Patient demographics and baseline characteristics.

Patient Characteristics	62 Eyes (46 Patients)
Mean age, years (SD)	71.6 (8.9)
Sex Female, n (%)	25 (54.3)
Mean DM duration, years (SD)	25.0 (12.1)
DM type, n (%) Type 1 Type 2	7 (15.2) 39 (84.8)
Mean DME duration, months (SD)	60.3 (30.6)
Mean follow-up duration, months (SD) Eyes followed, n (%) >6 months >12 months >18 months >24 months	13.9 (7.5) 50 (80.6) 38 (61.3) 24 (38.7) 4 (0.6)
Mean baseline IOP, mmHg (SD)	13.4 (2.7)
Mean HbA1c, % (SD)	7.9 (1.3)
Pseudophakic eyes, n (%)	60 (96.8)
History of vitrectomy, n (%)	18 (29.0)
Bilateral Fac injections, n (%)	16 (34.8)
Mean baseline BCVA, ETDRS letters (SD)	64.0 (17.2)
Mean baseline CMT, µm (SD)	333.3 (112.5)
Mean number of previous intravitreal injections, n (SD)	16.0 (9.1)
Anti-VEGF Ranibizumab Aflibercept Bevacizumab	9.2 (6.0) in 52 eyes (83.9%) 6.4 (4.8) in 32 eyes (51.6%) 6.4 (3.7) in 42 eyes (67.7%) 2.0 (0.0) in 3 eyes (5%)
Corticosteroids Dexamethasone Triamcinolone	8.2 (4.4) in 62 eyes (100%) 8.2 (4.2) in 62 eyes (100%) 2.25 (1.0) in 4 eyes (6.5%)
Laser, n (%): All Focal PRP Focal and PRP	47 (75.8) 18 (29.0) 45 (72.6) 16 (25.8)
Baseline IOP-lowering therapy, n (%) None Monotherapy Dual therapy >Dual therapy History of IOP-lowering surgery Previous SLT	41 (66.1) 11 (17.7) 10 (16.1) 0 1 (1.6) 5 (8.1)

BCVA: best corrected visual acuity; CMT: central macular thickness; DM: diabetes mellitus; DME: diabetic macular edema; ETDRS: early treatment diabetic retinopathy study; IOP: intraocular pressure; PRP: panretinal photocoagulation; SD: standard deviation; SLT: selective laser trabeculoplasty.

**Table 2:** Baseline associated factors for additional treatment after FAc.

	Mean Final BCVA, ETDRS Letters (SD)	*p*-Value	Mean BCVA Gain, ETDRS Letters (SD)	*p*-Value
Baseline <50 letters 50–60 letters >60 letters	41.1 (15.0) letters 60.5 (14.7) letters 74.0 (8.0) letters	*p* < 0.001	9.7 (10.3) letters 3.6 (11.6) letters −0.5 (6.4) letters	*p* = 0.02
DME duration <24 months 24–48 months >48 months	65.3 (16.1) letters 77.2 (7.1) letters 62.1 (17.2) letters	*p* = 0.004	−2.2 (8.4) letters 3.5 (8.0) letters 2.1 (9.5) letters	*p* = 0.5
DME thickness <300 µm 300–400 µm >400 µm	60.6 (18.0) letters 70.6 (14.4) letters 69.5 (13.3) letters	*p* = 0.1	2.3 (9.4) letters −1.7 (7.2) letters 8.6 (8.1) letters	*p* = 0.01
History of vitrectomy Yes No	62.1 (15.4) letters 67.6 (16.7) letters	*p* = 0.1	0.3 (9.9) letters 2.6 (8.7) letters	*p* = 0.2
Presence of SD-OCT biomarkers, n (%) SRF IRF HRF DRIL EZ/IZ alterations Hard exudates	78.3 (5.8) letters 66.0 (16.6) letters 68.0 (15.2) letters 57.5 (21.5) letters 58.8 (16.4) letters 65.1 (17.7) letters	*p* = 0.2 *p* = 0.8 *p* = 0.01 *p* = 0.09 *p* < 0.001 *p* = 0.9	10.0 (0.0) letters 1.8 (9.0) letters 1.8 (8.9) letters 1.8 (10.3) letters 2.1 (10.5) letters 2.8 (10.4) letters	*p* = 0.048 *p* = 0.4 *p* = 0.6 *p* = 0.9 *p* = 0.9 *p* = 0.9

BCVA: best corrected visual acuity; CMT: central macular thickness; DME: diabetic macular edema; DRIL: disorganization of retinal inner layers; ETDRS: early treatment diabetic retinopathy study; EZ/IZ: ellipsoid/interdigitation zone; HRF: hyperreflective foci; IRF: intraretinal fluid; SD: standard deviation; SRF: subretinal fluid.

**Table 3:** Baseline associated factors for additional treatment after FAc.

	No Additional Treatment (N = 39)	Additional Treatment (N = 23)	*p*-Value
Mean baseline BCVA, letters (SD)	66.2 (17.1)	60.3 (17.1)	0.1
Mean baseline CMT, µm (SD)	308.5 (79.1)	375.3 (146.2)	0.2
Mean DM duration, years (SD)	27.6 (12.4)	22.6 (12.9)	0.1
Mean DME duration, months (SD)	62.3 (34.2)	56.9 (23.5)	0.5
History of PRP, n (%)	24 (61.5)	21 (91.3)	0.01
History of vitrectomy, n (%)	9 (23.1)	9 (39.1)	0.2
Baseline SD-OCT biomarkers, n (%): SRF IRF HRF DRIL EZ/IZ alterations Hard exudates	2 (5.1) 36 (92.3) 34 (87.2) 12 (30.8) 21 (53.8) 7 (17.9)	1 (4.3) 23 (100.0) 22 (95.7) 3 (13.0) 18 (78.3) 6 (26.1)	0.7 0.3 0.4 0.1 0.06 0.3

BCVA: best corrected visual acuity; CMT: central macular thickness; DM: diabetes mellitus; DME: diabetic macular edema; DRIL: disorganization of retinal inner layers; ETDRS: early treatment diabetic retinopathy study; EZ/IZ: ellipsoid/interdigitation zone; HRF: hyperreflective foci; IRF: intraretinal fluid; PRP: panretinal photocoagulation SD: standard deviation; SRF: subretinal fluid.

**Table 4:** Intraocular pressure outcomes.

	N (%)
**Parameters after FAc** Δ IOP < 6 mmHg (low responder) Δ IOP 6–15 mmHg (intermediate responder) Δ IOP > 15 mmHg (high responder)	43 (69.4%) 17 (27.4%) 2 (3.2%)
IOP > 21 mmHg IOP > 25 mmHg IOP > 30 mmHg IOP > 25 mmHg and/or gain > 10 mmHg (OHT) IOP > 30 mmHg and/or gain > 15 mmHg	6 (9.7%) 4 (6.5%) 1 (1.6%) 7 (11.3%) 2 (3.2%)
**IOP procedures after FAc:** All Introduction of monotherapy Introduction of dual therapy Switch monotherapy to dual therapy Switch dual therapy to quadruple therapy SLT only Total of SLT IOP-lowering surgery following topical therapy	11 (17.7%) 2 (3.2%) 2 (3.2%) 4 (6.5%) 1 (1.6%) 2 (3.2%) 4 (6.5%) 1 (1.6%)

BCVA: best corrected visual acuity; CMT: central macular thickness; DM: diabetes mellitus; DME: diabetic macular edema; DRIL: disorganization of retinal inner layers; ETDRS: early treatment diabetic retinopathy study; EZ/IZ: ellipsoid/interdigitation zone; HRF: hyperreflective foci; IRF: intraretinal fluid; PRP: panretinal photocoagulation SD: standard deviation; SRF: subretinal fluid.

## Data Availability

All data can be obtained upon request to the corresponding author.
